# Mental Barriers and Links Connecting People of Different Cultures: Experiential vs. Conceptual Bases of Different Types of the WE-Concepts

**DOI:** 10.3389/fpsyg.2015.01950

**Published:** 2015-12-22

**Authors:** Maria Jarymowicz

**Affiliations:** Faculty of Psychology, University of WarsawWarsaw, Poland

**Keywords:** belonging to social groups *vs*. categories, interpersonal similarities, common values

There are numerous determinants of closeness in relationships between people. Cognitive representations of others in one's own mind are considered especially meaningful (Cooley, 1902; Durkheim, 1953; Markus and Moya, 2010). In particular, openness to others depends on the types of mental links between the Self and Others, leading to the formation of the WE concept.

The WE concept has specific affective and behavioral components. Some studies have demonstrated it in a spectacular way. Brewer and Gardner (1996) found that the number of instances of the word “we” used in neutral texts correlated with the lower or higher degree of further conformist reactions in participants who read given text. The simple “we” label (used in neutral context), associated in the mind with social context, stimulated behavior oriented toward social expectations.

The WE concept connects people, but at the same time can be the basis for powerful barriers and, sometimes, hostility. Empirical findings have shown the significance of the WE–THEY mental differentiation. Using *the minimal group paradigm*, psychologists demonstrated that even artificial groups (identified based on trivial criteria) displayed differentiation of in-group vs. out-group attitudes, especially evident in-group favoritism (Billig and Tajfel, 1973; Brewer, 1979; Tajfel, 1981, 1982). Even preschool children display out-group discrimination (Bar-Tal, 1996). In our studies (Jarymowicz, 2004), similarly to adults, the 9–10 years old participants displayed the *IAT effect* (Greenwald et al., 1998), connecting Pols rather with “flowers” and Germen rather with “insects.” However, people are able to develop mental representations in such a way as to be able to separate cognitive differentiation from evaluative discrimination.

## Social belonging and social categorizations

The desire for interpersonal attachment and the need to belong are considered basic determinants for human attitudes and choices (Schachter, 1959; Baumaister and Leary, 1995). However, it should be emphasized that social belonging concerns two different types of human communities (Tajfel, 1981) which should not be confused. As individuals, we belong to social groups *vs*. social categories—small communities with real, direct contacts, common goals, and actions, and to large categories (teachers, women, or Europeans), in which majority of members are linked “only” conceptually.

The first type of belonging, rooted in face-to-face interaction, leads to strong identification with in-group members, bolstered by material and psychological interdependence. The second type refers to larger social categories in which majority of members are reciprocally unknown. Identification is based on the mental links between the Self and Others and a feeling of belonging, which lead to positive attitudes (even toward those who as individuals would stimulate antipathy). The WE concept is constructed in opposition to THEY. Social psychologists paid a lot of attention to the fact that such WE—THEY opposition automatically led to social categorizations and discrimination (Billig and Tajfel, 1973; Fiske and Neuberg, 1989; Abrams and Hogg, 1999; Kwiatkowska, 1999; Grzesiak-Feldman, 2006).

Social categories (as a reference point for the need for identity gratification) often have blurred boundaries, and frequently cannot be easily distinguished from one another. The result is uncertainty and the need to determine who does and who does not belong to the WE category (who is a “true” Pole, German or Jew). Consequently, the perception of the WE homogeneity and WE–THEY difference is often significantly overestimated (Suls and Miller, 1977; Snyder and Fromkin, 1980; Fiske and Neuberg, 1989; Brewer, 1993). Moreover, THEY are identified based on oversimplified stereotypes and prejudices (Allport and Kramer, 1946; Campbell, 1967), often associated with negative emotions (Brewer, 1979; Tajfel, 1982). In extreme cases, such factors raise fundamental obstacles to the so-called *intractable conflicts* resolution (Bar-Tal, 2013).

## Beyond social belonging: Common personal attributes and moral values as the basis of the we concept

As social beings, we associate the WE concepts mainly with our belonging to social groups and categories. However, we are able to develop other types of WE concepts through conscious consideration of ourselves and others. These reflections can proceed by social comparisons or by comparing the “real” self and others to abstract (ideal) evaluative standards.

Within interpersonal comparisons we may find similarities with members of different social categories in terms of particular personal attributes. The WE can be referred to people (of different sex, age, race or nationality) who—as myself—“are involved in defending human rights” or “enjoy techno-music.” Social identity can be bridge across diverse social categories, based on interpersonal similarities.

Additionally, the Self can be connected with Others in abstract ways: by reference to ideal standards and moral concepts (Reykowski, 1979; Higgins, 1987; Jarymowicz, 2012; Jarymowicz and Szuster, 2014; Jarymowicz and Imbir, 2015). In this case, a mental encounter between the SELF and the OTHER can lead to a new type of the WE concept, namely to the formula of “WE as people,” brought together by the realization that all people form a universal community.

With that reasoning in mind (Jarymowicz, 1998, 2008), we identified four types of WE concepts (associated with different forms of social identity): (1) WE as a group of individuals (the group identity), (2) WE as a social category of people (the categorial identity), (3) WE as people of different social backgrounds, with common attributes (the attributive identity), (4) WE as human beings (the axiological identity).

Three of the above categories (attributive identity included) are associated with conditional acceptance of OTHERS: they are accepted if they belong to the same social group or category, or if they have some personal attributes (traits, attitudes or aims) similar to one's own. Only the axiological identity can be result from unconditional acceptance of others.

## Some empirical evidence for the relevance of the we types differentiation

In empirical studies we used the *Questionnaire of Social Perception* (Jarymowicz, 1994) to distinguish subjects with different types of the WE concept. In response to the open question “Who are the people I call WE?,” participants are asked to enumerate as many answers as they would like to. These answers are scored by the experimental team, and participants are assigned to groups with different dominant WE concept type—based on the proportion of a given type of answers. In this procedure there are almost no answers related to universal WE concept. Thus, we usually compared three “WE groups”: (1) WGR: WE as a group (answers like “Myself and my family,” “Our basketball team”), (2) WCT: WE as a category (e.g., “We, nurses,” “We, Poles”), (3) WAT: WE as people with similar attributes (e.g., “We, optimists,” “We, environmentalists”). In some other studies (Grzesiak-Feldman, 2006; Jarymowicz, 2006), the different types of WE were stimulated through experimental manipulation: participants read one of several short texts in which an unknown individual presented his/her social identifications. Pointed out were such sentences as: in WGR conditions “I spend a lot of time with my school friends. We go out every weekend …,” in the WCT conditions “For me, the most important thing is to maintain relations with people of my profession. I read journals edited by our association…,” and in WAT conditions “I feel close to people who are nonconformist, empathetic, optimistic. When I travel in Poland or abroad, I am drawn toward such people ….” In all studies we compared the above groups and conditions with regard to their attitudes toward strangers.

In the studies of Kwiatkowska (1999), participants were requested to estimate the usefulness of faces in guessing strangers' social belonging (like nationality, religion). As predicted, such estimations of importance of physical attributes were significantly higher among the WCT than among the WGR participants. This result is consistent with the assumption that categorial identity prompts social categorizations even if their basis is ambiguous, unsound, vague, and trivial (Fiske and Neuberg, 1989). It does not concern such a social identity form which is clearly related to a particular group membership: related to the WE as a group. In some studies we used implicit and explicit stimuli denoting different nationalities, and indirect attitudes measures. Data confirmed our predictions: participants with attributive identity displayed significantly more favorable attitudes toward strangers than those in the remaining categories (Szuster, 2005). In a long series of studies Grzesiak-Feldman (2006) found that belief in conspiracy theory (Jewish conspiracy) was significantly more prevalent in WCT condition (“WE as a social category”) than in the WGR one (“WE as a group”), but even more so compared with WAT conditions (“WE as people of similar preferences, hobbies, or interests”).

In the framework of our studies on the axiological identity we realized that this stage of the development of social identifications is much more difficult to achieve. The concept refers to certain intellectual capacities which we consider crucial for developing a sense of community among people across diverse cultures. Of those skills, understanding of abstract concepts associated with moral values seems especially important. With such psychological foundation it is possible, for example, to defend human rights even of those whom we perceive as intellectually or ethically inferior. To recognize some axiological predispositions we pay attention to the measurements of the so-called *axiological complexity*, which permit a subject to find out a meaning of abstract concepts like *honesty, loyalty* or *tolerance*, and so-called *axiological emotionality*, related to affective components of such concepts which influence probability of development of personal standards based on such superior values (Jarymowicz, 2010).

In empirical studies we describe the axiological complexity in terms of a number of real life referents one is able to list (regardless of their validity which can't be precisely assessed), simply as a measure of the one's readiness for moral reflection. Thus, we ask participants to generate (as many as possible) manifestations of *justice, righteousness, humanism* (etc.). To measure the axiological emotionality, we use the classic version of the Osgood's semantic differential (Osgood et al., 1957): participants have to confront abstract concepts like *tolerance* or *liberty* with words related to sensual pleasure/displeasure (like *sweet–bitter*, or *hard–soft*). The primary results show (Jarymowicz, 2010, 2012; Karwowska and Kobylinska, 2014) that the higher the number of indicated values referents and degree of positivity of affective components of axiological concepts, the lower the influence of implicit affective priming, the lower acceptance of anti-axiological social norms (like death penalty), and the higher indices of favorable attitudes toward social minorities.

## Conclusions

Empirical findings seem to support important claims and assumptions. (1) The concept “WE as a group” has a solid base in a given community and a real experience in the course of direct contacts, thus to maintain own identity any cognitive biases nor simplified stereotypes of the outgroup members are not needed. (2) The concept “WE as a social category” has to be carefully distinguished from the previous one, since the successful search of one's own social identity needs the “intergroup” (properly: intercategorial) cognitive and evaluative discrimination. (3) The attributive WE concept can be developed regardless of social belonging, as a consequence of cross-categorial, interpersonal perception of common personal traits—leading to feeling of community even with the “out-group” members. (4) The concept “WE as people, as human beings” (related to so-called axiological identity) can be developed through the reflective references of the Self and the Others to abstract moral values, which make possible to accept others regardless of their social status, and (in some conditions) lead to emotional identification with strangers.

### Conflict of interest statement

The author declares that the research was conducted in the absence of any commercial or financial relationships that could be construed as a potential conflict of interest.
